# Acute Multiple Cerebral Watershed Infarctions as the Initial Presentation of COVID-19 in a Young Male Patient: A Case Report

**DOI:** 10.7759/cureus.15119

**Published:** 2021-05-19

**Authors:** Randa Abdelmasih, Ramy Abdelmaseih, Radhames Ramos De Oleo

**Affiliations:** 1 Internal Medicine, University of Central Florida College of Medicine, Ocala, USA

**Keywords:** covid 19, stroke, weakness, neurological deficit

## Abstract

Cerebrovascular diseases appear to be one of the most serious complications of coronavirus disease 2019 (COVID-19). In this report, we present a case of a 38-year-old male with a past medical history significant only for hypertension, who presented to the emergency department (ED) with confusion and multiple focal neurologic deficits. Brain imaging showed acute multiple cerebral watershed infarctions. Upon further investigation and laboratory workup, the hypercoagulability and vasculitis panels were found to be negative, and other differential diagnoses were ruled out. In light of a number of emerging reports of COVID-19-related ischemic stroke, our patient was also screened for the disease, and surprisingly the test came back positive. We believe this case report will highlight the importance of conducting neurological examinations in COVID-19 patients, since timely workup and prompt interventions may reduce morbidity and mortality.

## Introduction

The novel coronavirus disease 2019 (COVID-19) pandemic has been regarded as the most severe global public health crisis since the influenza outbreak in 1918. Apart from its various common manifestations, it has also been associated with hypercoagulability that leads to life-threatening cardiovascular and neurovascular complications. The pathogenesis of this predisposition is not well understood, but one of its consequences is an increased risk of stroke. Patients with patent foramen ovale (PFO), atrial fibrillation, previous history of stroke, and elderly patients with uncontrolled hypertension are probably at a higher risk of developing stroke due to COVID-19. We present a rare case of acute multiple cerebral watershed infarctions in a COVID-19 patient who did not present with any of the aforementioned risk factors. Our main objective is to highlight the clinical presentation and the proposed mechanisms of neurovascular involvement in COVID-19.

## Case presentation

**Figure 1:**
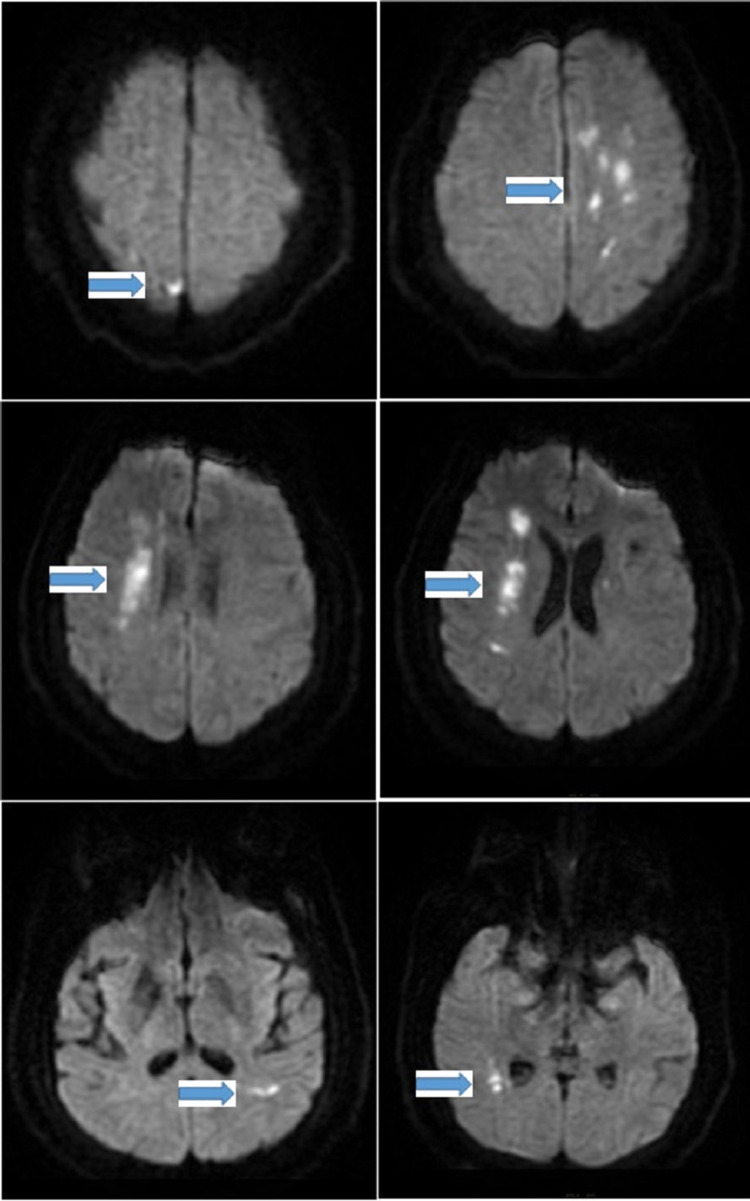
MRI brain showing multiple bilateral patchy small areas of acute infarctions related to watershed distribution (arrows) MRI: magnetic resonance imaging

**Figure 2:**
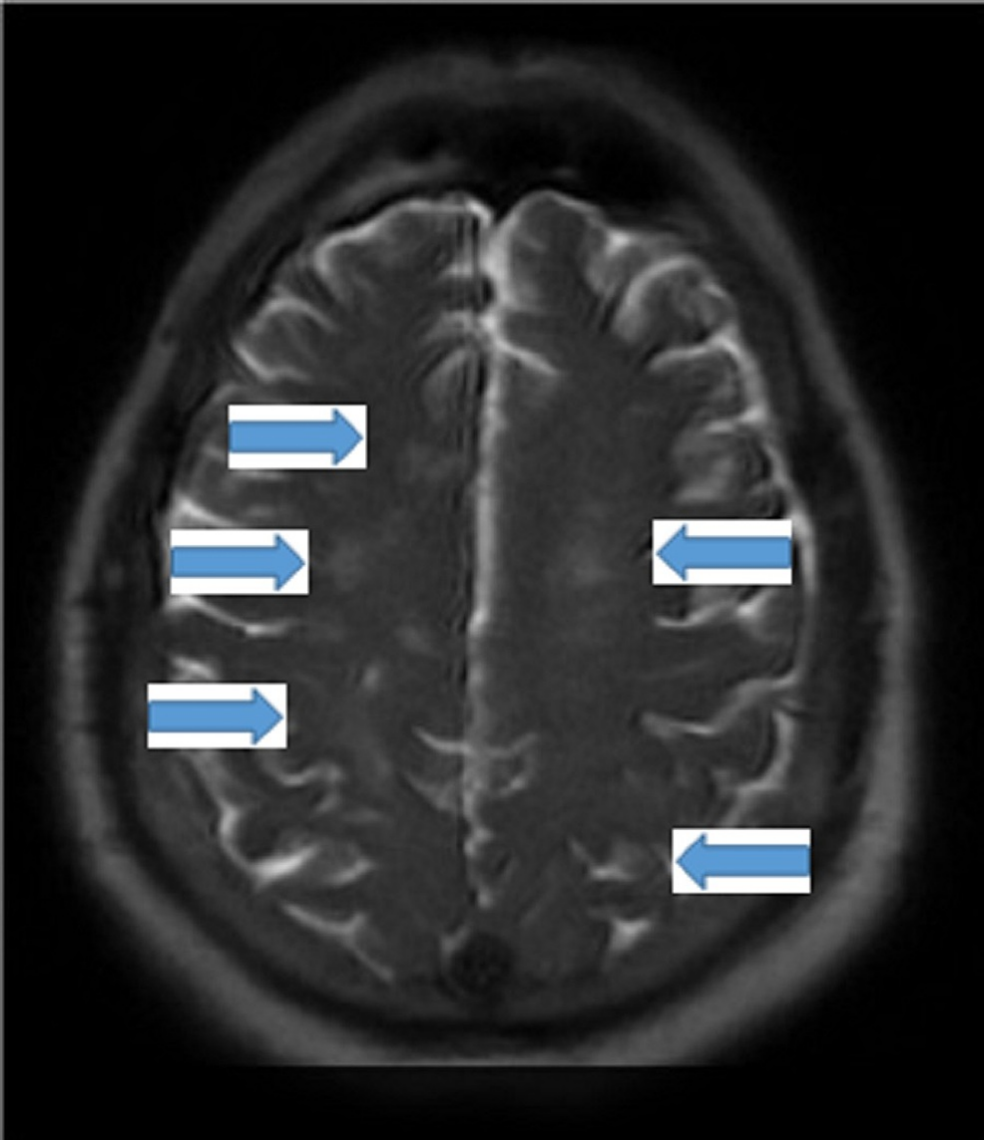
MRI brain showing multiple bilateral patchy acute infarctions (arrows) MRI: magnetic resonance imaging

A 38-year-old obese male with a past medical history of hypertension presented to the hospital with confusion, left-sided upper and lower extremity weakness, right lower extremity weakness, and right-gaze preference. CT scan and MRI of the brain demonstrated multiple patchy small areas of acute ischemic infarcts bilaterally consistent with watershed infarcts (Figures 1, 2). An echocardiogram with bubble study was negative for PFO. Lower extremity venous Doppler ultrasound was negative for deep venous thrombosis (DVT). Telemetry did not record any arrhythmias. Laboratory workup was negative for autoimmune diseases, hypercoagulable states, and vasculitis including antiphospholipid antibodies, lupus anticoagulants, anti-neutrophil cytoplasmic antibodies, anti-nuclear antibodies, anti-factor Xa, anti-thrombin, protein S and C, and rapid plasma regain. The patient was screened for COVID-19 in the context of increased reports of stroke in young patients nationwide, and surprisingly, the test was positive. Subsequently, aspirin, clopidogrel, high-intensity statins, and enoxaparin for DVT prophylaxis were initiated.

## Discussion

COVID-19 has turned into a major healthcare emergency of global concern. Evidence increasingly shows that it is not always confined to the respiratory tract but can also induce neurologic diseases with associated increased risk of stroke in young patients [1,2]. The reported incidence of stroke in COVID-19-positive patients is about 1-6% [3]. Although not completely understood, the plausible proposed mechanism of cerebrovascular disease in COVID-19 includes viral neurotropism, coagulopathy from systemic inflammation and cytokine storm, and endothelial dysfunction leading to angiopathic thrombosis. There have been a handful of cases linking COVID-19 to large vessel macrothrombosis and antiphospholipid syndrome [4]. We reported an even more unusual case that presented with multiple focal neurologic symptoms and was found to have watershed infarctions secondary to COVID-19 with only mild respiratory symptoms, and without typical vascular risk factors, PFO or arrhythmia, after excluding autoimmune diseases, hypercoagulability diseases, and vasculitis; this report highlights the importance of suspecting this devastating disease in an otherwise healthy young patient who presents with focal neurological symptoms.

## Conclusions

In COVID-19 patients with mild respiratory symptoms, a low threshold for investigation for stroke should be adopted if any new neurological symptoms are observed.
